# Molecular docking studies of salubrinal and its analogs as inhibitors of the GADD34:PP1 enzyme

**DOI:** 10.5599/admet.632

**Published:** 2019-04-05

**Authors:** Pavlo V. Zadorozhnii, Ihor O. Pokotylo, Vadym V. Kiselev, Oxana V. Okhtina, Aleksandr V. Kharchenko

**Affiliations:** Department of Organic Substances and Pharmaceutical Preparations, Ukrainian State University of Chemical Technology, Gagarin Ave., 8, Dnipro 49005, Ukraine

**Keywords:** salubrinal, molecular docking, GADD34:PP1, RMSD, endoplasmic reticulum stress

## Abstract

The phenomenon of the endoplasmic reticulum (ER) stress as a molecular pathophysiological process underlies diseases as cancer, diabetes mellitus, myocardial infarction, neurodegenerative disorders, diseases of the urinary system, disorders associated with bone integrity, etc. To prevent ER stress, salubrinal, which is a phosphatase inhibitor of the eukaryotic translation initiation factor - GADD34:PP1, is currently being intensively studied. The aim of this work is to search for new analogues of this drug using molecular docking methods. Optimization of the geometry of the studied structures and molecular docking was carried out using the ArgusLab 4.0.1 software package. The three-dimensional crystal structure of the GADD34: PP1 enzyme (PDB ID: 4XPN) was loaded in the PDB format from the protein molecule data bank. The model of the binding site was created on the basis of the phosphoric acid residue (403 PO4). The dimensions of the binding site were set manually and were 40.000 Å along the X-axis, 40.000 Å - the Y-axis and 40.000 Å - the Z-axis. The docking was done with a flexible ligand, and the semi-empirical AScore function was used for the scoring procedure. It was shown that for the salubrinal molecule the most favorable was the conformation stabilized by the intramolecular hydrogen bond formed between the hydrogen atom of the thiourea fragment and the oxygen atom of the amide fragment. According to molecular docking data, six compounds from the fifty-four analyzed analogues of salubrinal exceed it in the stability of the complex formed with GADD34:PP1. The results of this work can be used to create new phosphatase inhibitors of the eukaryotic translation initiation factor GADD34:PP1.

## Introduction

Endoplasmic reticulum (ER) is an intracellular membrane organelle that is extremely sensitive to changes in homeostasis. The membrane ER is integrated with the cell nucleus membrane. The internal ER space opens directly into the perinuclear space, which accompanies the contact of the ER signalling device with the genetic material. There are granular (rough) ER and agranular (smooth) ER. Smooth ER is located on the periphery of the organelle and is responsible for the synthesis of lipids, steroids, the metabolism of carbohydrates, medicines and other exogenous products [1,2].

Rough ER is an extension of the cell nucleus membrane. On its cytosolic surface, ribosomes are deposited, which provide for the translation of the protein directly into the ER cavity through the system of transmembrane channels. Inside the granular ER, “immature” protein molecules are foldable, i.e. take a correct spatial conformation. All unfolded or incorrectly folded proteins are caught and necessarily destroyed. Accumulation of misfolded protein molecules results in a functional overload of ER. This phenomenon is called ER stress and it leads to disorders in the normal functioning of the cell and threatens it with death [1,2].

Over the past 15 years [3], molecular mechanisms of ER stress have been intensively studied as a fundamental phenomenon of cell protection from the action of various factors and as a molecular pathophysiological process leading to many severe diseases [3-21].

**Figure 1.**
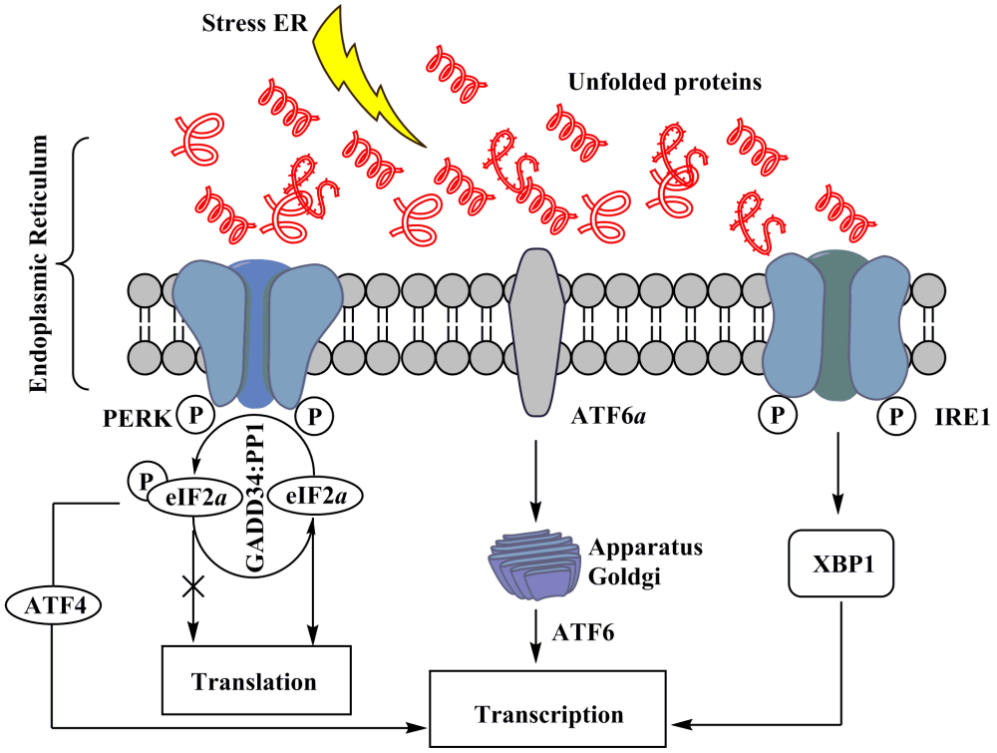
Schematic simplified image of the main signal-sensory systems of ER stress

Figure 1 schematically depicts the response of the cell to ER stress, which is necessary for the cell to find ways to escape from the state of stress caused by the accumulation of unfolded or misfolded proteins, and which is mediated by three signal-sensory systems that begin in the ER lumen and terminate in the cytoplasm and nucleus [22-26]. Induction of ER stress stops the penetration of synthesized proteins into it and accompanies both the proper folding of proteins, which are already in it and the degradation of misfolded ones. This is necessary for the survival of the cell under the conditions of the factors that induce this stress, or death of the cell through the apoptosis system associated with ER [27,28].

The main signal-sensory ER stress systems (PERK, ATF6 and IRE1), which originate in its lumen under the conditions of accumulation of unfolded or incorrectly folded proteins in it, initiate total repression of translation initiation by phosphorylation of eukaryotic translation initiation factor 2α (eIF2α), and activation of the transcription of stress dependent genes by the formation of an active form of transcription factors ATF4 and ATF6, as well as an alternative splice variant of the transcription factor XBP1 (X-Box Protein-1), which controls the expression of the cell genes [19,26].

In this way, the response of cells to ER stress, which is mediated by the three signal-sensory systems, is necessary for the cell to find possible ways out of the state of stress caused by the accumulation of unfolded or incorrectly folded proteins in the ER lumen.

EIF2α is a key participant in protein translation because it is responsible for binding the 40S ribosomal subunit to tRNA_imet_ (initiation of methionine tRNA), which recognizes the mRNA start codon and starts the synthesis of the peptide chain [19]. PERK phosphorylates eIF2α translating it into an inactive eIF2αP form. However, the holoenzyme complex GADD34: PP1 dephosphorylates eIF2αP, again translating it into an active eIF2α form.

**Figure 2.**
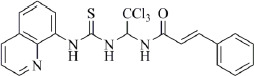
Structure of the salubrinal molecule

In 2005 M. Boyce and colleagues reported that salubrinal (Fig. 2) acted as a phosphatase inhibitor GADD34:PP1, selective for eukaryotic translation initiation factor 2α (eIF2α) [29]. Thus, salubrinal weakens the synthesis of unfolded or misfolded proteins contributing to the preservation of homeostasis in ER and saving cells from apoptosis.

Since the beginning of intensive studies of salubrinal, its protective effect has been confirmed in a number of studies [30]. Although salubrinal is currently under development, we can already say with certainty about its prospects in the treatment of diabetes [31], myocardial infarction [32], neurodegenerative disorders [33,34], oncological diseases [35], diseases of the genitourinary system [36] and disorders related to the integrity of bone tissue [37,38]. Work is underway to study its toxicity and the development of analogues [39].

In this paper, using the methods of molecular docking [40,41], we have established the binding site of the salubrinal preparation with holoenzyme GADD34:PP1 and searched for analogues of this drug.

## Materials and methods

### Computer specification

All calculations were carried out on a Toshiba personal computer, the Satellite L650D model, AMD Phenom(tm) II P820 Triple-Core Processor. A 64-bit operating system was used.

### Ligand preparation

The search for structures for research was conducted in the SciFinder database (https://scifinder.cas.org) (see supporting information). Prior to molecular docking, the structures of all the compounds studied were optimized within the semiempirical PM3 method [42] using the ArgusLab 4.0.1 software package [43-47]. The calculation of the electron density distribution in the static salubrinal molecule was carried out with the ZINDO approximation method [48] in the same software package.

### Protein preparation

The three-dimensional crystal structure of the GADD34:PP1 enzyme (4XPN) was loaded in the PDB format from the protein molecules data bank (http://www.rcsb.org). Prior to docking, the molecules of all the non-proteinaceous components, except for one phosphoric acid residue, having the code in co-crystallisate 403 PO4, were removed. Hydrogen atoms were added throughout the protein structure before molecular docking.

### Molecular docking procedure

Based on the phosphoric acid residue (403 PO4), a ligand group was created with the given name Ligand_X-ray. Based on this group, a three-dimensional model of a binding site was created, the dimensions of which were set manually and amounted along the X-axis – 40.000 Å, the Y-axis – 40.000 Å and the Z-axis – 40.000 Å. Docking was performed with a flexible ligand. For the scoring procedure, the semi-empirical function AScore was used created on the basis of the XScore function [49]. The resolution of the cell was set at 0.250 Å. The calculation type was Dock; Docking Engine - ArgusLab. Visualization of the results was carried out using the program PyMOL [50].

## Results and discussion

### The results of ligand geometry optimization

**Figure 3.**
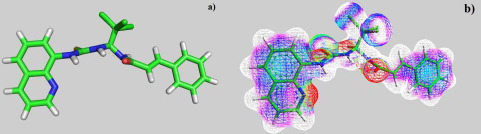
a) The calculated structure of the salubrinal molecule (PM3 method), visualization in PyMol; b) Distribution of electron density in the static salubrinal molecule. Colors: a) □ 0.0500 → 0.0409; b) → 0.0409 → 0.0318; c) → 0.0318 → 0.0227; d) → 0.0227 → 0.0136; e) → 0.0136 → 0.0045; f) → 0.0045 → -0.0045; g) → -0.0045 → -0.0136; h) → -0.0136 → -0.0227; i) → -0.0227 → -0.0318; j) → -0.0318 → -0.0409; k) → -0.0409 → -0.0500

According to the results of optimization of the geometry of the salubrinal molecule, the most stable is the conformation stabilized by the intramolecular hydrogen bond formed between the hydrogen atom of the thiourea fragment and the oxygen atom of the amide fragment (Fig. 3a). The length of the NH...O=C bond is 1.891 Å. That is, the salubrinal molecule exists as a pseudo 1,3,5-oxadiazine ring with an angle H...O=C 108.33 °. According to X-ray diffraction data for 1,3,5-oxadiazine cycles, this angle is somewhat larger and lies within the range of 114.76-120.00° [51-53]. The appearance of an intramolecular hydrogen bond is obviously associated with a large difference in the static charges on the oxygen atom of the amide fragment and the hydrogen atom of the thiourea fragment. According to calculations of the electron density distribution in the static salubrinal molecule (the ZINDO approximation method), on the oxygen atom δ^-^ lies within -0.0409→-0.0500, in turn, on the hydrogen atom δ^+^ is 0.0500→0.0409 (Fig. 3b). The presence of an intramolecular hydrogen bond is characteristic of all the salubrinal analogues studied (*see* Supporting information, Tables S1 and S2).

### The results of molecular docking

**Figure 4.**
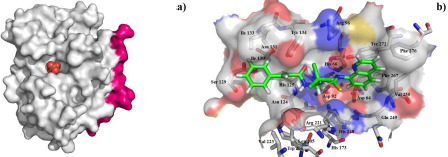
a) The structure of the GADD34:PP1 holoenzyme. Protein phosphatase 1 (PP1) is represented in white color, and GADD34 - pink light. In the active site of the holoenzyme, there is a phosphoric acid residue, depicted in the form of spheres; b) the orientation of the *salubrinal* molecule in the active site of the GADD34:PP1 holoenzyme according to molecular docking data.

The active center of selective dephosphatase of the eukaryotic translation initiation factor (Fig. 4a) contains the phosphoric acid residue, and two Mg^2+^ ions (not shown in the figure). In the active site of the GADD34:PP1, it is possible to distinguish three sites, one hydrophilic - located approximately in its center, and two lipophilic ones located on the periphery. Therefore, the interactions of the salubrinal molecule with the active site of the GADD34:PP1 enzyme are represented by both polar contacts (Fig. 4b) and lipophilic interactions between the cinnamic acid residue, the quinoline ring and the lipophilic regions of the active site. The molecule of salubrinal effectively interacts with the GADD34:PP1 enzyme closing access to the active site. The energy of the complex GADD34:PP1-salubrinal forms -12.2489 kcal/mol. The salubrinal molecule is additionally fixed in the active center of the enzyme due to the formation of an intermolecular hydrogen bond involving the amino acid Tyr 272 (Fig. 4b). A hydrogen bond arises between the nitrogen atom of the pyridine type of the quinoline ring and the hydroxyl group of Tyr 272 (the length of the N...HO bond is 3.432 Å). The salubrinal molecule is also fixed due to the shortened intermolecular polar contacts: 1) between the oxygen atom of the amide fragment and the hydroxyl group Tyr 134; 2) between the sulfur atom of the thiourea fragment and the guanidine fragment Arg 221.

**Figure 5.**
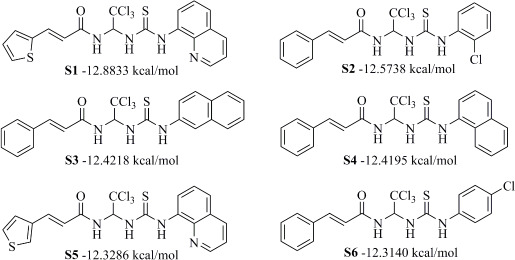
Structures of salubrinal analogues, surpassing it in the strength of the salubrinal preparation superior to the strength of the formed complex with the GADD34:PP1 holoenzyme.

To determine the effect of the residue of cinnamic acid and quinoline cycle in the salubrinal preparation on the ability to bind to the active site of the GADD34:PP1 enzyme, we periodically replaced one of the fragments with other groups. According to the molecular docking data, six compounds out of fifty-four analyzed salubrinal analogues (*see supporting information Table S3 and S4*) exceeded it in the stability of the complex formed with GADD34:PP1 (Fig. 5).

**Figure 6.**
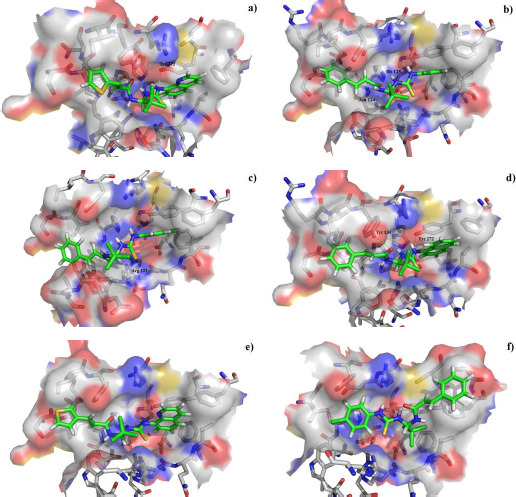
Position of the molecules of the compounds (**S1**)-(**S6**) in the active center of the GADD34:PP1 holoenzyme.

The most stable complex with GADD34:PP1 is formed by (*E*)-3-(thiophen-2-yl)-*N*-(2,2,2-trichloro-1-(3-(quinolin-8-yl)thioureido)ethyl)acrylamide (**S1**) (Fig. 6a), the energy of the complex with GADD34:PP1 is -12.8833 kcal/mol, RMSD 1.4 Å. The molecule of the compound (**S1**) is additionally fixed in the enzyme active site due to the intermolecular hydrogen bond formed between the nitrogen atom of the thiourea fragment and the -OH group of Tyr 272, the length of the HN...HO bond is 3.605 Å). It is also fixed due to the formation of shortened intermolecular polar contacts: 1) between the oxygen atom of the amide fragment and the hydroxyl group of Tyr 134; 2) between the sulfur atom of the thiourea fragment and the guanidine fragment Arg 221.

The energy of the complex *N*-(2,2,2-trichloro-1-(3-(2-chlorophenyl)thioureido)ethyl)cinnamamide (**S2**) with GADD34:PP1 forms -12.5738 kcal/mol, RMSD 2.3 Å (Fig. 6b). The molecule of the compound (**S2**) is additionally fixed in the active site of the enzyme due to the formation of two intermolecular hydrogen bonds involving amino acids His 125 and Asn 124 (Fig. 6b). Both hydrogen bonds are formed by the oxygen atom of the amide fragment. In the first case, the hydrogen bond is with the pyrrole atom of nitrogen of the imidazole ring His 125 (the C=O...HN bond length is 2.133 Å), and in the second case - with the amide fragment of amino acid Asn 125 (the C=O...H_2_NC(O) bond length is 2.884 Å). The molecule of the compound (**S2**) is also fixed due to the shortened intermolecular polar contact between the sulfur atom of the thiourea fragment and the guanidine fragment Arg 221.

*N*-(2,2,2-Trichloro-1-(3-(naphthalen-2-yl)thioureido)ethyl)cinnamamide (**S3**) forms the complex with the GADD34:PP1 enzyme having the energy of -12.4218 kcal/mol, RMSD 1.4 Å (Fig. 6c). The molecule of the compound (**S3**) is additionally stabilized in the enzyme active center due to the intermolecular hydrogen bond formed by the oxygen atom of the oxygen amide fragment and the HN group of Tyr Arg 221, the C=O...HN bond length is 2.481 Å. The molecule of the compound (**S3**) is also stabilized due to the shortened intermolecular polar contact between the sulfur atom of the thiourea fragment and the guanidine fragment Arg 221.

The energy of the complex *N*-(2,2,2-trichloro-1-(3(naphthalene-1-yl)thioureido)ethyl)cinnamamide (**S4**) with GADD34:PP1 forms -12.4195 kcal/mol, RMSD 4.8 Å (Fig. 6d). The molecule of the compound (**S4**) is additionally stabilized in the enzyme active center due to the formation of intermolecular hydrogen bonds: 1) between the oxygen atom of the amide fragment and the -OH group of Tyr 134, the C=O...HO bond length is 2.758 Å; 2) between the nitrogen atom of the thiourea fragment and the -OH group of Tyr 272, the HN...HO bond length is 2.999 Å. Moreover, stabilization occurs due to the shortened intermolecular polar contact between the sulfur atom of the thiourea fragment and the guanidine fragment Arg 221.

(*E*)-3-(Thiophen-3-yl)-*N*-(2,2,2-trichloro-1-(3-(quinolin-8-yl)thioureido)ethyl)acrylamide **(S5)** and *N*-(2,2,2-trichloro-1-(3-(4-chlorophenyl)thioureido)ethyl)cinnamamide **(S6)** form complexes with the GADD34:PP1 enzyme having the energy of -12.3286 kcal/mol (RMSD - 1.3 Å) and -12.3140 kcal/mol (RMSD - 5.4 Å), respectively (Fig. 6e, 6f). The compounds (**S5**) and (**S6**) do not form intermolecular hydrogen bonds in the active center of GADD34:PP1, their interaction with amino acids forming the active site is obviously hydrophobic in nature. In this case, the formation of weak shortened intermolecular polar contacts is possible, for example, between the sulfur of the thiourea fragment and the guanidine fragment Arg 221. It should be noted, that the molecule of the compound (**S6**) in the active center of GADD34:PP1 is rotated 180° as compared to the salubrinal molecule and the remaining compounds hits.

**Figure 7.**
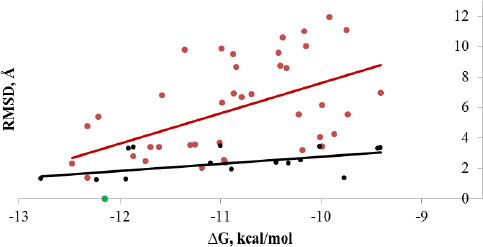
Energy dependence of the complex GADD34:PP1-Inhibitor on the RMSD value. Color: a) → quinoline derivatives; → cinnamic acid derivatives; → salubrinal, taken into account when constructing a linear regression line in both cases.

Figure 7 shows that for both quinoline derivatives and cinnamic acid derivatives, the energy of the complex formed is clearly related to GADD34:PP1 from the RMSD value. The quinoline derivatives interact closely with the active site of the enzyme, the RMSD value does not exceed 3.5 Å. While for the cinnamic acid derivatives, RMSD can vary from 1.5 to 12.0 Å. This is due to the fact that there are two lipophilic sites in the active center of the enzyme. The quinoline cycle, due to spatial difficulties, is clearly fixed only in one of them, and the cinnamic acid residue can interact with both. This can lead to a reversal of the inhibitor molecule located in the active center by 180° relative to the salubrinal molecule, which is observed, for example, for the compound (**S6**).

Based on our findings, when searching for the GADD34:PP1 inhibitors, other than cinnamic acid and quinoline derivatives, special attention should be paid to the compounds containing a naphthalene and isoquinoline ring, heterocyclic analogs of cinnamic acid, and compounds containing chlorine atoms in the aromatic ring. The results of our work are in good agreement with the already published experimental data on establishing the dependence of the structure-activity of salubrinal analogues [39,54]. For example, the low activity of 2-amino-pyridine derivatives, for which the EC_50_ lies in the range of 28-72 μM [39], as compared to the derivatives of 8-aminoquinoline (EC_50_ = 15-16 μM) [39,54], can be explained by the high energy of the complex that they form with GADD34:PP1. The lower energy of the GADD34:PP1-Inhibitor complex can also explain the high efficiency of (*E*)-3-(thiophen-2-yl)acrylamide derivatives (EC_50_ = 4-43 μM) compared with cinnamamide derivatives (EC_50_ = 6-57 μM) [54].

## Conclusions

In this paper, the search for new analogues of salubrinal has been carried out by molecular modeling. We have shown that the most stable conformation of the salubrinal molecule and its analogues contains the intramolecular hydrogen bond between the hydrogen atom of the thiourea fragment and the oxygen atom of the amide fragment. The binding site of salubrinal to the active site of the enzyme has been established. We have found the compounds, which form stronger complexes with the enzyme than salubrinal itself. The results of this work can be used to create new phosphatase inhibitors of the eukaryotic translation initiation factor GADD34:PP1.

**Supporting Information for the paper:** Molecular docking studies of salubrinal and its analogs as inhibitors of the GADD34:PP1 enzyme

**Table S1.** The results of geometry optimization of salubrinal analogues containing cinnamic acid residue 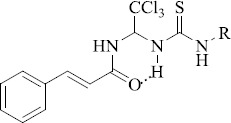

Entry	R	CAS No	*E*, kcal/mol	The length of the NH...O=C bond, Å	Angle value С=O…H, degrees	Time, s
1	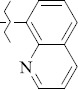	405060-59-9	-111285.9025	1.891	108.33	347
2	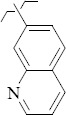	863036-22-0	-111285.2829	1.880	108.88	348
3	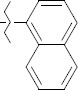	294654-78-7	-110631.9704	2.273	105.11	344
4	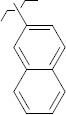	324769-18-8	-110634.7191	1.863	108.63	365
5	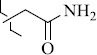	405060-99-3	-98255.3837	1.919	109.47	192
6	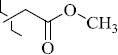	405060-96-0	-104370.9745	1.883	108.96	207
7	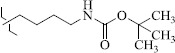	863036-35-5	-129157.9187	1.943	108.52	736
8	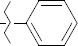	301359-85-3	-99001.5764	1.891	108.31	232
9	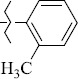	301359-95-5	-102453.0936	1.863	108.48	272
10	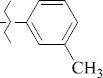	301359-86-4	-102454.5158	1.864	108.24	240
11	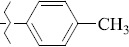	301359-87-5	-102454.6059	1.868	108.08	249
12	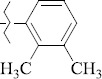	1429483-71-5	-105904.3082	1.863	108.55	315
13	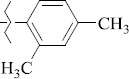	301359-93-3	-105905.3171	1.865	108.40	288
14	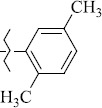	301359-94-4	-105905.2465	1.861	108.55	287
15	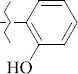	294657-79-7	-105777.6924	1.858	107.98	247
16	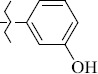	3037775-31-7	-105778.1081	1.890	108.48	271
17	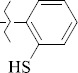	405060-94-8	-103291.5220	1.863	108.80	246
18	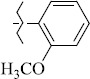	301359-88-6	-109214.6095	1.874	108.24	288
19	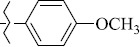	1346508-38-0	-109215.6380	1.886	108.42	298
20	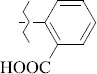	303775-35-5	-115287.0750	1.858	108.47	295
21	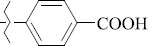	294655-14-4	-115289.4152	1.870	108.75	318
22	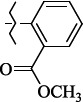	294655-12-2	-118722.8463	1.858	108.12	359
23	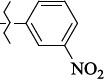	294654-81-2	-115870.4210	1.871	108.65	291
24	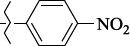	294654-82-3	-115870.3348	1.868	108.55	301
25	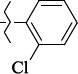	301359-89-7	-105946.6228	1.864	108.36	241
26	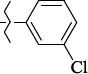	301359-90-0	-105947.3592	1.860	108.50	224
27	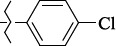	301359-91-1	-105947.5373	1.859	108.49	234
28	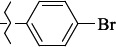	301359-92-2	-106797.8334	1.860	108.54	237
29	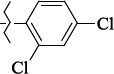	301359-97-7	-112891.5613	1.862	108.63	254
30	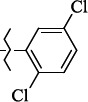	301359-98-8	-112891.2835	1.863	108.60	277
31	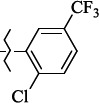	301359-96-6	-138810.5173	1.859	108.45	355
32	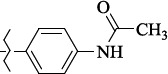	301815-13-4	-116057.8543	1.863	108.53	375
33	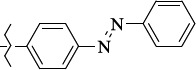	294653-17-1	-124258.2307	1.896	108.75	723
34	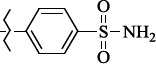	294654-77-6	-120926.7177	1.862	108.67	409
35	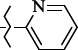	863036-23-1	-99655.3253	1.857	108.48	215
36	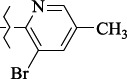	1349267-41-9	-110899.9614	1.873	108.85	294
37	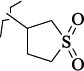	1349267-41-9	-112093.4153	2.339	104.89	272
38	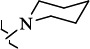	1346508-37-9	-97718.9301	2.507	105.22	233
39	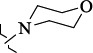	1346508-36-8	-101035.0119	2.521	105.03	225
40	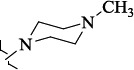	1429483-72-6	-101809.4188	2.493	105.51	275

**Table S2.** The results of geometry optimization of salubrinal analogues containing quinoline ring 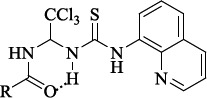

Entry	R	CAS No	E, kcal/mol	The length of the NH...O=C bond, Å	Angle value С=O…H, degrees	Time, seconds
1	CH_3_-	294658-37-0	-90772.1333	1.835	109.78	141
2	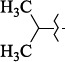	324769-75-5	-97666.2121	1.849	109.08	207
3	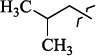	294658-28-9	-101115.4287	1.832	109.23	261
4	(CH_3_)_3_C-	412962-51-7	-101113.4907	1.843	109.50	247
5	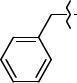	305856-11-5	-108568.4619	2.370	101.87	319
6	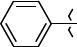	294646-80-3	-105112.9884	2.387	101.67	291
7	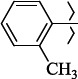	324017-95-0	-108568.5668	2.460	101.65	323
8	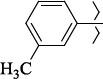	330684-99-6	-108574.0942	2.261	105.45	330
9	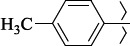	294658-30-3	-108574.2167	2.418	105.03	313
10	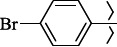	330567-60-7	-112916.8710	2.288	104.53	308
11	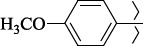	294658-45-0	-115333.8079	2.382	102.98	363
12	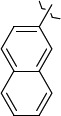	405060-98-2	-116754.1849	2.262	104.94	437
13	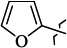	324017-57-4	-105701.1794	2.347	102.79	246
14	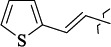	1346508-21-1	-109395.6421	1.901	108.14	336
15	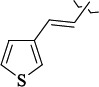	1346508-22-2	-109398.3840	1.913	107.96	313

**Table S3.** The results of molecular docking of salubrinal analogues containing cinnamic acid residue 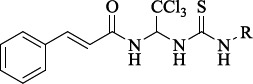

Entry	R	CAS No	E, kcal/mol	RMSD, Å	Time, seconds
1	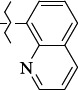	405060-59-9	-12.2489	-	68
2	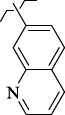	863036-22-0	-11.4506	9.8	64
3	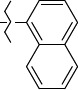	294654-78-7	-12.4195	4.8	66
4	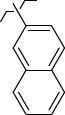	324769-18-8	-12.4218	1.4	68
5	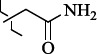	405060-99-3	-10.4440	8.6	222
6	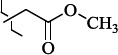	405060-96-0	-10.9403	8.7	712
7	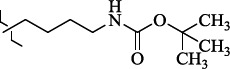	863036-35-5	-11.0821	6.3	25055
8	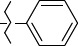	301359-85-3	-11.0494	2.4	91
9	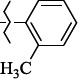	301359-95-5	-11.8415	2.5	78
10	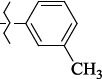	301359-86-4	-11.7071	3.4	81
11	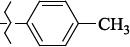	301359-87-5	-10.9713	9.5	82
12	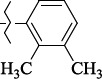	1429483-71-5	-11.4002	3.5	70
13	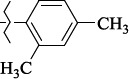	301359-93-3	-11.0643	2.6	74
14	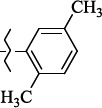	301359-94-4	-10.7865	6.86	70
15	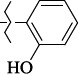	294657-79-7	-11.9634	2.8	76
16	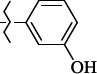	3037775-31-7	-10.8881	6.7	80
17	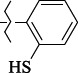	405060-94-8	-11.2845	2.0	75
18	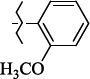	301359-88-6	-11.3522	3.6	152
19	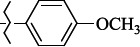	1346508-38-0	-9.8336	5.5	182
20	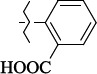	303775-35-5	-11.7954	3.4	176
21	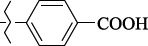	294655-14-4	-10.0874	3.4	202
22	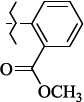	294655-12-2	-11.1046	3.7	501
23	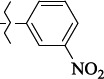	294654-81-2	-9.9688	4.2	201
24	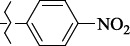	294654-82-3	-10.2628	11.0	195
25	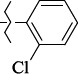	301359-89-7	-12.5738	2.3	86
26	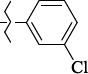	301359-90-0	-10.2843	3.2	88
27	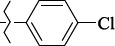	301359-91-1	-12.3140	5.4	88
28	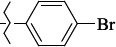	301359-92-2	-10.4781	10.6	87
29	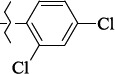	301359-97-7	-10.5242	9.6	82
30	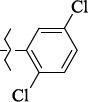	301359-98-8	-11.0901	9.8	77
31	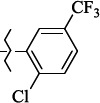	301359-96-6	-10.5026	8.7	140
32	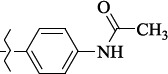	301815-13-4	-10.9633	6.9	178
33	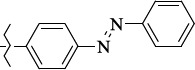	294653-17-1	-11.6777	6.8	45
34	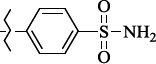	294654-77-6	-10.2446	10.0	108
35	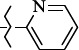	863036-23-1	-9.8478	11.1	87
36	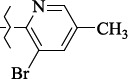	1349267-41-9	-10.0146	11.9	60
37	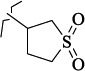	1349267-41-9	-9.5065	6.9	60
38	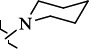	1346508-37-9	-10.1080	4.1	35
39	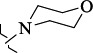	1346508-36-8	-10.0909	6.1	35
40	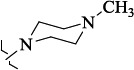	1429483-72-6	-10.3209	5.5	31

**Table S4.** The results of molecular docking of salubrinal analogues containing quinoline ring 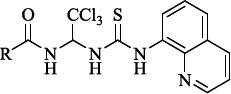

Entry	R	CAS No	E, kcal/mol	RMSD, Å	Time, seconds
1	CH_3_-	294658-37-0	-9.5079	3.4	30
2	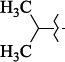	324769-75-5	-9.5363	3.3	37
3	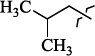	294658-28-9	-10.1110	3.4	48
4	(CH_3_)_3_C-	412962-51-7	-10.5443	2.4	35
5	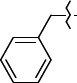	305856-11-5	-11.0971	3.5	55
6	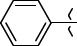	294646-80-3	-12.0377	1.3	38
7	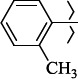	324017-95-0	-12.0111	3.3	34
8	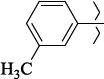	330684-99-6	-10.2993	2.6	37
9	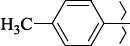	294658-30-3	-10.9900	1.9	36
10	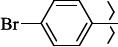	330567-60-7	-11.9627	3.4	36
11	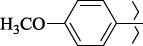	294658-45-0	-10.4252	2.4	54
12	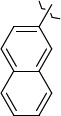	405060-98-2	-11.1993	2.4	35
13	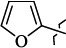	324017-57-4	-9.8686	1.4	38
14	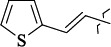	1346508-21-1	-12.8833	1.4	74
15	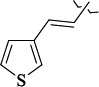	1346508-22-2	-12.3286	1.3	75

## References

[1] Schwarz D.S. Blower M.D.. The endoplasmic reticulum: structure, function and response to cellular signaling. Cell Mol. Life Sci. 73 (2016) 79-4. doi:10.1007/s00018-015-2052-6. 10.1007/s00018-015-2052-6 26433683 PMC4700099

[2] English A.R. Voeltz G.K.. Endoplasmic Reticulum Structure and Interconnections with Other Organelles. Cold. Spring. Harb. Perspect. Biol. 5 (2013) a013227. doi: 10.1101/cshperspect.a013227. 10.1101/cshperspect.a013227 23545422 PMC3683900

[3] Blumental-Perry A.. Endoplasmic Reticulum Stress Response, the Future of Cancer Research and a New Designated Journal. Endoplasmic Reticulum Stress in Cancers 1 (2013) 1-3. doi:10.2478/ersc-2012-0001. 10.2478/ersc-2012-0001

[4] Schönthal A.H.. Endoplasmic Reticulum Stress: Its Role in Disease and Novel Prospects for Therapy. Scientifica (2012) 26 pages. doi: 10.6064/2012/857516. 10.6064/2012/857516 PMC3820435 24278747

[5] Hetz C.. The unfolded protein response: controlling cell fate decisions under ER stress and beyond. Nat. Rev. Mol. Cell Biol. 13 (2012) 89-102. doi:10.1038/nrm3270. 10.1038/nrm3270 22251901

[6] Oakes S.A. Papa F.R.. The role of endoplasmic reticulum stress in human pathology. Annu. Rev. Pathol. 10 (2015) 173-194. doi:10.1146/annurev-pathol-012513-104649. 10.1146/annurev-pathol-012513-104649 25387057 PMC5568783

[7] Ozcan L. Tabas I.. Role of endoplasmic reticulum stress in metabolic disease and other disorders. Annu. Rev. Med. 63 (2012) 317-328. doi:10.1146/annurev-med-043010-144749. 10.1146/annurev-med-043010-144749 22248326 PMC3290993

[8] Hosoi T. Ozawa K.. Endoplasmic reticulum stress in disease: mechanisms and therapeutic opportunities. Clin. Sci. (Lond) 118 (2009) 19-29. doi:10.1042/CS20080680. 10.1042/CS20080680 19780718

[9] Gardner B.M. Pincus D. Gotthardt K. Gallagher C.M. Walter P.. Endoplasmic reticulum stress sensing in the unfolded protein response. Cold. Spring. Harb. Perspect. Biol. 5 (2013) a013169. doi:10.1101/cshperspect.a013169. 10.1101/cshperspect.a013169 23388626 PMC3578356

[10] Bhandary B. Marahatta A. Kim H.-R. Chae H.-J.. An Involvement of Oxidative Stress in Endoplasmic Reticulum Stress and Its Associated Diseases. Int. J. Mol. Sci. 14 (2013) 434-456. doi:10.3390/ijms14010434. 10.3390/ijms14010434 PMC3565273 23263672

[11] Pagliassotti M.J.. Endoplasmic Reticulum Stress in Nonalcoholic Fatty Liver Disease. Annu. Rev. Nutr. 32 (2012) 17-33. doi:10.1146/annurev-nutr-071811-150644. 10.1146/annurev-nutr-071811-150644 22809102

[12] Cybulsky A.V.. Endoplasmic reticulum stress, the unfolded protein response and autophagy in kidney diseases. Nat. Rev. Nephrol. 13 (2017) 681-696. doi:10.1038/nrneph.2017.129. 10.1038/nrneph.2017.129 28970584

[13] Navid F. Colbert R.A.. Causes and consequences of endoplasmic reticulum stress in rheumatic disease. Nat. Rev. Rheumatol. 13 (2017) 25-40. doi:10.1038/nrrheum.2016.192. 10.1038/nrrheum.2016.192 27904144

[14] Urra H. Dufey E. Avril T. Chevet E. Hetz C.. Endoplasmic Reticulum Stress and the Hallmarks of Cancer. Trends. Cancer. 2 (2016) 252-262. doi:10.1016/j.trecan.2016.03.007. 10.1016/j.trecan.2016.03.007 28741511

[15] Colgan S.M. Al-Hashimi A.A. Austin R.C.. Endoplasmic reticulum stress and lipid dysregulation. Expert. Rev. Mol. Med. 13 (2011) e4. doi:10.1017/S1462399410001742. 10.1017/S1462399410001742 21288373

[16] Sprenkle N.T. Sims S.G. Sánchez C.L. Meares G.P.. Endoplasmic reticulum stress and inflammation in the central nervous system. Mol. Neurodegener. 12 (2017) 42. doi: 10.1186/s13024-017-0183-y. 10.1186/s13024-017-0183-y 28545479 PMC5445486

[17] Omura T. Kaneko M. Okuma Y. Matsubara K. Nomura Y.. Endoplasmic Reticulum Stress and Parkinson’s Disease: The Role of HRD1 in Averting Apoptosis in Neurodegenerative Disease. Oxid. Med. Cell Longev. (2013) 7 pages. doi:10.1155/2013/239854. 10.1155/2013/239854 PMC3654363 23710284

[18] Eizirik D.L. Miani M. Cardozo A.K.. Signalling danger: endoplasmic reticulum stress and the unfolded protein response in pancreatic islet inflammation. Diabetologia 56 (2013) 234-241. doi:10.1007/s00125-012-2762-3. 10.1007/s00125-012-2762-3 23132339

[19] Chistiakov D.A. Sobenin I.A. Orekhov A.N. Bobryshev Y.V.. Role of endoplasmic reticulum stress in atherosclerosis and diabetic macrovascular complications. Biomed. Res. Int. (2014) 14 pages. doi:10.1155/2014/610140. 10.1155/2014/610140 PMC4100367 25061609

[20] Meyerovich K. Ortis F. Allagnat F. Cardozo A.K.. Endoplasmic reticulum stress and the unfolded protein response in pancreatic islet inflammation. J. Mol. Endocrinol. 57 (2016) R1-R17. doi:10.1530/JME-15-0306. 10.1530/JME-15-0306 27067637

[21] Xiang C. Wang Y. Zhang H. Han F.. The role of endoplasmic reticulum stress in neurodegenerative disease. Apoptosis 22 (2017) 1-26. doi:10.1007/s10495-016-1296-4 10.1007/s10495-016-1296-4 27815720

[22] Marciniak S.J. Ron D.. Endoplasmic reticulum stress signaling in disease. Physiol. Rev. 86 (2006) 1133-1149. doi:10.1152/physrev.00015.2006. 10.1152/physrev.00015.2006 17015486

[23] McQuiston A. Diehl J.A.. Recent insights into PERK-dependent signaling from the stressed endoplasmic reticulum. F1000Res. 6 (2017) 11 pages. doi:10.12688/f1000research.12138.1. 10.12688/f1000research.12138.1 PMC5664976 29152224

[24] Schwarz D.S. Blower M.D.. The endoplasmic reticulum: structure, function and response to cellular signaling. Cell Mol. Life Sci. 73 (2016) 79-94. doi:10.1007/s00018-015-2052-6. 10.1007/s00018-015-2052-6 26433683 PMC4700099

[25] Pluquet O. Pourtier A. Abbadie C.. The unfolded protein response and cellular senescence. A review in the theme: cellular mechanisms of endoplasmic reticulum stress signaling in health and disease. Am. J. Physiol. Cell Physiol. 308 (2015) C415-425. doi:10.1152/ajpcell.00334.2014. 10.1152/ajpcell.00334.2014 25540175

[26] Kadowaki H. Nishitoh H.. Signaling Pathways from the Endoplasmic Reticulum and Their Roles in Disease. Genes (Basel) 4 (2013) 306-333. doi:10.3390/genes4030306. 10.3390/genes4030306 24705207 PMC3924831

[27] Sano R. Reed J.C.. ER stress-induced cell death mechanisms. Biochim. Biophys. Acta 1833 (2013) 3460-3470. doi:10.1016/j.bbamcr.2013.06.028. 10.1016/j.bbamcr.2013.06.028 23850759 PMC3834229

[28] Iurlaro R. Muñoz-Pinedo C.. Cell death induced by endoplasmic reticulum stress. FEBS J. 283 (2016) 2640-2652. doi:10.1111/febs.13598. 10.1111/febs.13598 26587781

[29] Boyce M. Bryant K.F. Jousse C. Long K. Harding H.P. Scheuner D. Kaufman R.J. Ma D. Coen D.M. Ron D. Yuan J.. A Selective Inhibitor of eIF2α Dephosphorylation Protects Cells from ER Stress. Science 307 (2005) 935-939. doi:10.1126/science.1101902. 10.1126/science.1101902 15705855

[30] Matsuoka M. Komoike Y.. Experimental Evidence Shows Salubrinal, an eIF2α Dephosphorylation Inhibitor, Reduces Xenotoxicant-Induced Cellular Damage. Int. J. Mol. Sci. 16 (2015) 16275-16287. doi:10.3390/ijms160716275. 10.3390/ijms160716275 26193263 PMC4519949

[31] Cnop M. Ladriere L. Hekerman P. Ortis F. Cardozo A.K. Dogusan Z. Flamez D. Boyce M. Yuan J. Eizirik D.L.. Selective inhibition of eukaryotic translation initiation factor 2alpha dephosphorylation potentiates fatty acid-induced endoplasmic reticulum stress and causes pancreatic beta-cell dysfunction and apoptosis. J. Biol. Chem. 282 (2007) 3989-3997. doi:10.1074/jbc.M607627200. 10.1074/jbc.M607627200 17158450

[32] Liu Y. Wang J. Qi S.Y. Ru L.S. Ding C. Wang H.J. Zhao J.S. Li J.J. Li A.Y. Wang D.M.. Reduced endoplasmic reticulum stress might alter the course of heart failure via caspase-12 and JNK pathways. Can. J. Cardiol. 30 (2014) 368-375. doi:10.1016/j.cjca.2013.11.001. 10.1016/j.cjca.2013.11.001 24565258

[33] Sokka A.L. Putkonen N. Mudo G. Pryazhnikov E. Reijonen S. Khiroug L. Belluardo N. Lindholm D. Korhonen L.. Endoplasmic reticulum stress inhibition protects against excitotoxic neuronal injury in the rat brain. J. Neurosci. 27 (2007) 901-908. doi:10.1523/JNEUROSCI.4289-06.2007. 10.1523/JNEUROSCI.4289-06.2007 17251432 PMC6672923

[34] Zhu Y. Fenik P. Zhan G. Sanfillipo-Cohn B. Naidoo N. Veasey S.C.. Eif-2a Protects Brainstem Motoneurons in a Murine Model of Sleep Apnea. J. Neurosci. 28 (2008) 2168-2178. doi:10.1523/JNEUROSCI.5232-07.2008. 10.1523/JNEUROSCI.5232-07.2008 18305250 PMC6671854

[35] Lee S.K. Kim Y.S.. Phosphorylation of eIF2α attenuates statin-induced apoptosis by inhibiting the stabilization and translocation of p53 to the mitochondria. Int. J. Oncol. 42 (2013) 810-816. doi: 10.3892/ijo.2013.1792. 10.3892/ijo.2013.1792 23354132 PMC3597453

[36] Katsoulieris E. Mabley J.G. Samai M. Green I.C. Chatterjee P.K.. α-Linolenic acid protects renal cells against palmitic acid lipotoxicity via inhibition of endoplasmic reticulum stress. Eur. J. Pharmacol. 623 (2009) 107-112. doi:10.1016/j.ejphar.2009.09.015. 10.1016/j.ejphar.2009.09.015 19765573

[37] He L. Lee J. Jang J.H. Sakchaisri K. Hwang J. Cha-Molstad H.J. Kim K.A. Ryoo I.J. Lee H.G. Kim S.O. Soung N.K. Lee K.S. Kwon Y.T. Erikson R.L. Ahn J.S. Kima B.Y. Osteoporosis regulation by salubrinal through eIF2α mediated differentiation of osteoclast and osteoblast. Cell. Signal. 25 (2013) 252-260. doi:10.1016/j.cellsig.2012.11.015. 10.1016/j.cellsig.2012.11.015 PMC3593652 23178987

[38] Hamamura K. Nishimura A. Iino T. Takigawa S. Sudo A. Yokota H.. Chondroprotective effects of salubrinal in a mouse model of osteoarthritis. Bone Joint. Res. 4 (2015) 84-92. doi:10.1302/2046-3758.45.2000378. 10.1302/2046-3758.45.2000378 25977571 PMC4443296

[39] Liu J. He K.L. Li X. Li R.J. Liu C.L. Zhong W. Li S.. SAR, Cardiac Myocytes Protection Activity and 3D-QSAR Studies of Salubrinal and its Potent Derivatives. Curr. Med. Chem. 19 (2012) 6072-6079. doi:10.2174/0929867311209066072. 10.2174/0929867311209066072 23036152

[40] Young D.C.. Computational drug design. John Wiley & Sons, Inc, New Jersey, USA 2009.

[41] Holtje H.-D. Sippl W. Rognan D. Folkers R.. Molecular Modeling. Basic Principles and Applications. Wiley-VCH Verlag GmbH & Co. KGaA, Weinheim, Germany, 2008.

[42] Thiel W.. Semiempirical quantum-chemical methods. Wiley Interdisciplinary Reviews: Computational Molecular Science 4 (2014) 145-157. doi:10.1002/wcms.1161. 10.1002/wcms.1161

[43] Thompson M.A. Zerner M.C.. A theoretical examination of the electronic structure and spectroscopy of the photosynthetic reaction center from *Rhodopseudomonas viridis*. J. Am. Chem. Soc. 113 (1991) 8210-8215. doi:10.1021/ja00022a003. 10.1021/ja00022a003

[44] Thompson M.A. Glendening E.D. Feller D.. The Nature of K^+^/Crown Ether Interactions: A Hybrid Quantum Mechanical-Molecular Mechanical Study. J. Phys. Chem. 98 (1994) 10465-10476. doi:10.1021/j100092a015. 10.1021/j100092a015

[45] Thompson M.A. Schenter G.K. Excited States of the Bacteriochlorophyll b Dimer of *Rhodopseudomonas viridis*: A QM/MM Study of the Photosynthetic Reaction Center That Includes MM Polarization. J. Phys. Chem. 99 (1995) 6374-6386. doi:10.1021/j100017a017. 10.1021/j100017a017

[46] Thompson M.A.. QM/MMpol: A Consistent Model for Solute/Solvent Polarization. Application to the Aqueous Solvation and Spectroscopy of Formaldehyde, Acetaldehyde, and Acetone. J. Phys. Chem. 100 (1996) 14492-14507. doi:10.1021/jp960690m. 10.1021/jp960690m

[47] Thompson M.. ArgusLab 4.0.1. Planaria software LLC, Seattle, Wash, USA, 2004. http://www.arguslab.com.

[48] Young D.C.. Computational Chemistry: A Practical Guide for Applying Techniques to Real World Problems. John Wiley & Sons, Inc, New York, USA, 2001. doi:10.1002/0471220655. 10.1002/0471220655

[49] Wang R. Lai L. Wang S.. Further development and validation of empirical scoring functions for structure-based binding affinity prediction. J. Comput. Aided. Mol. Des. 16 (2002) 11-26. 12197663 10.1023/a:1016357811882

[50] DeLano W.L.. The PyMOL Molecular Graphics System; DeLano Scientific: Palo Alto, CA, 2003; http://www.pymol.org .

[51] Onys’ko P.P. Sinitsa A.A. Pirozhenko V.V. Chernega A.N.. Synthesis of Phosphorylated 1,3,5-Oxadiazines via *N*-Acyltrifluoroacetimidoilphosphonates. Heteroatom. Chem. 13 (2002) 22-26. doi:10.1002/hc.1102. 10.1002/hc.1102

[52] Kennard K.K. Byriel K.A. Woon T.Ch. Fairlie D.P.. Structure of a novel protonated oxadiazine: an unusual heterocycle from the cycloaddition of a ketone with nitriles. Chem. Commun. 15 (1996) 1731-1732. doi:10.1039/CC9960001731. 10.1039/CC9960001731

[53] Zadorozhnii P.V. Kiselev V.V. Pokotylo I.O. Kharchenko A.V.. A new method for the synthesis of 4*H*-1,3,5-oxadiazine derivatives. Heterocycl. Commun. 23 (2017) 369-374. doi:10.1515/hc-2017-0083. 10.1515/hc-2017-0083

[54] Long K. Boyce M. Lin H. Yuan J. Ma D.. Structure-activity relationship studies of salubrinal lead to its active biotinylated derivative. Bioorg. Med. Chem. Lett. 15 (2005) 3849-52. doi:10.1016/j.bmcl.2005.05.120. 10.1016/j.bmcl.2005.05.120 16002288

